# Do Cultivated Varieties of Native Plants Have the Ability to Outperform Their Wild Relatives?

**DOI:** 10.1371/journal.pone.0071066

**Published:** 2013-08-12

**Authors:** Roland Schröder, Rüdiger Prasse

**Affiliations:** Leibniz Universität Hannover, Institute of Environmental Planning, Hannover, Germany; University of Tartu, Estonia

## Abstract

Vast amounts of cultivars of native plants are annually introduced into the semi-natural range of their wild relatives for re-vegetation and restoration. As cultivars are often selected towards enhanced biomass production and might transfer these traits into wild relatives by hybridization, it is suggested that cultivars and the wild × cultivar hybrids are competitively superior to their wild relatives. The release of such varieties may therefore result in unintended changes in native vegetation. In this study we examined for two species frequently used in re-vegetation (*Plantago lanceolata* and *Lotus corniculatus*) whether cultivars and artificially generated intra-specific wild × cultivar hybrids may produce a higher vegetative and generative biomass than their wilds. For that purpose a competition experiment was conducted for two growing seasons in a common garden. Every plant type was growing (a.) alone, (b.) in pairwise combination with a similar plant type and (c.) in pairwise interaction with a different plant type. When competing with wilds cultivars of both species showed larger biomass production than their wilds in the first year only and hybrids showed larger biomass production than their wild relatives in both study years. As biomass production is an important factor determining fitness and competitive ability, we conclude that cultivars and hybrids are competitively superior their wild relatives. However, cultivars of both species experienced large fitness reductions (nearly complete mortality in *L. corniculatus*) due to local climatic conditions. We conclude that cultivars are good competitors only as long as they are not subjected to stressful environmental factors. As hybrids seemed to inherit both the ability to cope with the local climatic conditions from their wild parents as well as the enhanced competitive strength from their cultivars, we regard them as strong competitors and assume that they are able to outperform their wilds at least over the short-term.

## Introduction

Vast amounts of cultivated varieties of native plants (hereafter cultivars) are annually introduced within the natural or semi-natural range of their wild relatives (hereafter wilds) for re-vegetation and restoration purposes. For instance, in Germany, approximately 13.700 tons of cultivated grass seeds and additionally 280 tons of cultivated herb seeds were imported during 2007 and 2008, from EU and non-EU countries (German Federal Office for Agriculture and Food, personal communication) and subsequently released into the environment. In the USA, 2.9 million ha of cultivars of native grass species were introduced only 2007 on former cropland through the USDA Conservation Reserve Program (USDA according to [1]).

The introduced cultivars may or may not establish successfully. Their establishment might fail if they are not adapted to the environmental conditions of the re-vegetation site. If the introduced individuals are adapted to the local environment, they may establish, coexist, and possibly hybridize with their wilds already established on the site. Hybridization is a common phenomenon if cultivars are introduced into the natural range of their wilds [2]. In some cases the introduced cultivars or evolving wild × cultivar hybrids (hereafter hybrids) may be competitively superior to their wilds. Subsequently, the introduced cultivars or the hybrids may replace their wilds [3]. The large-scale replacement of wild plants by cultivars or hybrids of the same species (we regard them as different plant types of a species) is undesirable for the conservation of native plants’ biodiversity. For instance, in parts of northern Spain (Galicia) cultivars of *Dactylis glomerata*, which were introduced for hay production in the 1970’s, and developed hybrids with their wilds seemed to displace the wild populations [4]. Similar trends have been assumed for *Lolium perenne* in Britain [5] and *L. multiflorum* in Switzerland [6] as cultivars of these species have been introduced for several decades to improve grasslands. This kind of displacements of wild plant populations is known as cryptic invasion because cultivars, wilds and their hybrids are often difficult to distinguish from another and the invasion is therefore not immediately detected [7], [8].

If cryptic invasion is detected, it is often unknown whether the cultivars or hybrids are really well established and can replace their wilds or whether their numerical dominance is a result of the permanent and strong propagule pressure (i.e. “seed-rain”) due to re-vegetation and restoration activities. The suspected competitive advantage of cultivars over their wilds may be due to the intensive “seed-rain” from re-vegetation activities (quantitative advantage) or due to changes in life-history traits or changes in morphology selected for during breeding and cultivation (qualitative advantage). For instance, Japanese *Ardisia crenata*, a shrub bred for its ornamental value, invaded North-American hardwood forests, likely due to a different leaf architecture, which enhanced its competitive ability [9]. Significant differences in plant architecture and fitness between cultivars and wilds have been observed for *Plantago lanceolata* and *Lotus corniculatus* (Schröder and Prasse, own observation), two species frequently used in re-vegetation in Central Europe. The cultivars exhibited longer leaves (*P. lanceolata*) and shoots (*L. corniculatus*), combined with a more vigorous and erect growth habit as well as a larger vegetative and generative biomass at the end of the growing season if compared to their wilds and if grown under constant human care (unheated PE cover-greenhouse, watered as needed). If it holds true that cultivars are selected towards vigorous growth and high biomass production, they may be competitively superior to their wilds due to differences in resource exploitation and/or resource allocation. Similar can be assumed for hybrids. Moreover, if hybrids exhibit heterosis in life-history traits relevant for competitive ability [10], a competitive superiority over their wilds will be increased.

Additionally, studies investigating which life-history traits may favor the invasion of non-native species often recorded positive relationships between biomass production and plant invasiveness along gradients of increasing resource availability [11], [12]. Thus, cultivars and hybrids might be especially superior competitors in productive habitats where competition for light is strong [13]. Taller cultivars and hybrids may have an advantage over smaller wilds as it is known that tall plants are often competitively superior to (e.g. [14], [15]) and may outcompete the smaller ones. Thus, species with similar life-history traits might coexist precisely because interspecific competition is approximately equal (or less) to intraspecific competition [16]. Based on that theory we assume that, if both cultivars and hybrids are competitive superior to their wild relatives, between-type competition (cultivar vs. wild, hybrid vs. wild) should be higher than within-type competition (wild vs. wild). Reversely, concerning cultivars and hybrids, between-type competition should be lower than within-type competition (cultivars vs. cultivar, hybrid vs. hybrid).

However, it is not known whether the permanent introduction of large amounts of cultivars of native plants might have unintended and undesirable effects on their wilds. As such knowledge is essential to evaluate the need for strategies in re-vegetation and plant production that mitigate the indicated problems, this study aims to test whether introduced cultivars as well as their hybrids with wilds are competetively superior to their wilds. We use aboveground biomass (vegetative and generative) as a parameter for competitive superiority because it has been shown that aboveground biomass production is a good indicator for competitive ability in productive habitats [17] and cultivars are selected for such productive environments as their production does not include limitations of nutrients and water. Additionally, we consider percentage survival as important factor of competitive superiority. In particular we tested the following hypotheses:

1. When competing with cultivars wilds produce less biomass than cultivars (between-type competition).
2. When competing with hybrids wilds produce less biomass than hybrids (between-type competition).
3. When competing with cultivars (between-type competition) wilds produce less biomass than wilds in competition with wilds (within-type competition).
4. When competing with hybrids (between-type competition) wilds produce less biomass than wilds in competition with wilds (within-type competition).
5. When competing with wilds (between-type competition) cultivars produce more biomass than cultivars in competition with cultivars (within-type competition).
6. When competing with wilds (between-type competition) hybrids produce more biomass than hybrids in competition with hybrids (within-type competition).
7. When competing with cultivars (between-type competition) wilds exhibit lower survival than wilds in competition with wilds (within-type competition).
8. When competing with hybrids (between-type competition) wilds exhibit lower survival than wilds in competition with wilds (within-type competition).

## Materials and Methods

### Selection of species

We selected *Plantago lanceolata* L. (Plantaginaceae) and *Lotus corniculatus* L. (Fabaceae) as study species, because both species are commonly cultivated in nurseries and used for re-vegetation in Central Europe and were able to get hold of cultivars as well as wilds. Both species are hemicryptophytes abundant in a wide range of habitats and distributed over large parts of the northern hemisphere. *P. lanceolata* is wind-pollinated and self-incompatible [18], [19]. *L. corniculatus* is an insect-pollinated and a predominantly outcrossing species [20].

### Origin of seed material

Seeds from two wild populations and two cultivars of *P. lanceolata* as well as from one wild population and one cultivar of *L. corniculatus* were used (Table 1). The wild seeds were collected from semi-natural grasslands near Hannover (52°26′16″ N, 9°46′41″ E, only *P. lanceolata*) and Celle (52°42′2″ N, 10°6′42″ E), Lower Saxony, Germany. The grasslands are located within military training areas and the responsible authority (Bundeswehr-Dienstleistungszentrum, Hannover) permitted the seed collections. The field collections did not affect endangered or protected species. Seeds were collected in wild populations where likely no seeds have been introduced for the last 60 years. A distance of at least 300 m was kept from adjacent populations where cultivars might have been introduced to reduce the chance for hybridization [21]. Seeds were collected as bulk sample from at least 200 individuals from each location in October 2008. The distance between sampled individuals was at least 5 m to collect a maximum of each population’s genetic variation. Only ripe seeds were collected directly from a mother plant (i.e. no seeds collected from soil surface; 3 spikes or pots per plant). The cultivars were obtained from a seed trading company (Feldsaaten Freudenberger GmbH & Co KG, Krefeld, Germany). We choose the cultivars of both species by ordering varieties from the seed trading company that are commonly used in re-vegetation (e.g. for landscaping) in Germany. The *L. corniculatus* variety is traded as “Gran San Gabriele” by the seed trading company and is selected for high productivity and resistance to cold.

**Table 1:** Plant types, origin and crossing schemes of studied species.

ID	Plant type	F1-generation crossing scheme	F0 origin
*Plantago lanceolata*			
1	Wild	Wild 1× Wild 1	Germany, Hannover
2		Wild 2× Wild 2	Germany, Celle
3	Cultivar	Cultivar 3× Cultivar 3	Hungary
4		Cultivar 4× Cultivar 4	Austria
5	Hybrid	Wild 1× Cultivar 3	–
6		Wild 1× Cultivar 4	–
7		Wild 2× Cultivar 3	–
8		Wild 2× Cultivar 4	–
*Lotus corniculatus*			
1	Wild	Wild × Wild	Germany, Celle
2	Cultivar	Cultivar × Cultivar	Uruguay
3	Hybrid	Wild × Cultivar	–

### Production of experimental plants

To minimize different maternal effects on plant fitness the experiment was conducted with individuals from the F1-generation where parental F0-plants were grown under identical environmental conditions. The germination of F0-seeds was initiated in February 2009 by treating *P. lanceolata* seeds with gibberellic acid (GA_3_ 500 ppm, 24 hours at 20°C and light), while *L. corniculatus* seeds were scarified with sandpaper (allowing water to penetrate the seed coat), to prevent undesirable pre-selection. Seeds were sown in potting soil (Hawita-Flor P+Ton: organic matter  = 60%, N = 150 mg/l, P_2_O_5_ = 150 mg/l, K_2_O = 200 mg/l) in a temperate greenhouse. The temperature was set to 20°C for a photoperiod of about 12 hour and 7°C for a 12 hour night. The solar irradiation in the greenhouse was similar to the solar irradiation under natural conditions (polyethylene cover-greenhouse). Germination success in *P. lanceolata* was above 90% and was about 80% in *L. corniculatus*. Thus, a large part of the genotypes represented by the seeds was available for the experiments. The F0-plants were separated into pots (0.5 l) with potting soil (Hawita-Flor T+Ton: organic matter  = 60%, N = 230 mg/l, P_2_O_5_ = 230 mg/l, K_2_O = 300 mg/l; 1∶1 mixture of sand and potting soil) two weeks after germination. From February 2009 until September 2009 the plants were grown in a randomized block design in an unheated PE cover-greenhouse and watered regularly and evenly.

F1-seeds were produced by artificially crossing during flowering season from June 2009 to August 2009. To produce hybrid seeds we used wild plants as pollen-acceptor and cultivated varieties as pollen-donator. For detailed description of cultivation and crossing methods see [22]. Consequently, we generated crossings of F1-types of wild × wild, cultivar × cultivar, and wild × cultivar (Table 1). For *P. lanceolata* we used 224 F0-plants (112 as male/112 as female) to generate 384 F1-plants. For *L. corniculatus* we used 84 F0-plants (42 as male/42 as female) to generate 162 F1-plants. For the competition experiment we used F1-seeds of the same number of maternal families of each single plant type (i.e. 14 maternal families per plant type) to avoid undesirable selection for strong reproductive genotypes and to ensure the use of same number of genotypes per plant type. The F1-generation seeds were treated as described above during April 2010 to grow plants for the experiment. The germination success was comparable to the success in 2009. Subsequently, seedlings were planted in multi-pot-palettes with one seedling per pot and grown in an unheated greenhouse (same as above) until they reached juvenile stadium (full development of primary leaves).

### Experimental set-up

The established juvenile F1-plants were potted in garden pots (7.5 l, Ø  = 25 cm) with potting soil (Hawita-Flor T+Ton: organic matter  = 60%, N = 230 mg/l, P_2_O_5_ = 230 mg/l, K_2_O = 300 mg/l; 1∶1 mixture of sand and potting soil) in April 2010. Each single plant type of a species was grown in two densities (i.e., alone and in pairwise competition). Specifically, each single plant type was grown (a.) alone (wild, cultivar or hybrid alone), (b.) in pairwise competition with a plant from the same plant type (wild with wild, cultivar with cultivar or hybrid with hybrid), as well as (c.) in pairwise competition with a plant from another plant type (wild with cultivar, wild with hybrid, or hybrid with cultivar). For *P. lanceolata* wilds, the between-type competition with hybrids was tested only with hybrid individuals of the particular half-sib family. Each treatment was replicated six times yielding a total of 186 pots (1 pot  =  one experimental unit) in *P. lanceolata* and 42 pots in *L. corniculatus*. Single plants (controls) were placed at the center of the pot. Plants in pairwise competition treatments were potted with a 9 cm distance to each other and each 8 cm distances to the pot edges. Pots were taken out to a common garden, arranged in a fully randomized design and recessed in-situ soil to reduce evaporation. Pots were weeded and watered regularly and evenly. The experiment was carried out for two growing seasons (April 2010 – November 2011).

### Measurements

Aboveground biomass was measured separately for vegetative and generative plant parts as both may contribute to competitive ability. Vegetative and generative dry biomass was evaluated twice, at the end of the first growing season in 2010 and at the end of the second growing season in 2011, each in November. The aboveground parts of all plants were clipped to 3 cm above soil surface. For *P. lanceolata*, generative parts of the plants (stipes with ripe spikes) were removed and stipes were counted. Dry weights of vegetative parts were determined for both study species by drying plant parts for 48 h at 85°C and subsequently weighting them with a 0.1 g balance. Evaluating generative biomass production for *P. lanceolata*, the averaged dry weight of ripe spikes were determined per individual, calculated from three randomly chosen spikes (without stipes), and multiplied with its total number of spikes. In *L. corniculatus* ripe pods were harvested before seed release daily during seasons, dried and weighted in the same way. Survival of individuals was recorded after each growing season.

### Data analysis

All statistical analyses were conducted using the computer program R 2.15.0 [23]. Analyses were carried out for each species separately, for each generation, and each year by sub-setting the dataset into growing season 2010 and growing season 2011. To account for possible data relatedness of single samples within similar plant types, for data of *P. lanceolata*, we fitted generalized linear mixed models (GLMM) with maximum likelihood [24] using the R package “arm” [25]. We fitted models on the datasets by Bayesian methods as this is the most appropriate method for testing GLMM [26]. We used the single samples (ID, Table 1) as random factor and plant type as fixed factor. Additionally, we fitted generalized linear models (GLM) with maximum likelihood for both species with plant type as independent factor. Response variable was always the parameter used as measurement for vegetative biomass or generative biomass. In *P. lanceolata* we used Akaike's information criterion (AIC) as a measure of the fit of the GLMM and GLM. For further analysis we used the model with the smaller AIC followed by model simplification (deletion of non-significant factors). Based on GLMM and GLM, respectively, posterior distributions of the parameters (i.e. response variables) as well as 95% credible intervals and parameter differences according to hypotheses 1–6 were estimated by carrying out 1000 independent simulations using the “sim”-function within the “arm”-package [27]. Additionally, we evaluated differences in biomass production by calculating posterior probabilities.

For the estimates of parameter differences we consider an effect significant if the posterior probability *p* is larger or equal to 0.95 and zero is not or narrowly included in the 95% Bayesian credible interval of an estimate. The limits of a 95% credible interval were obtained as the 2.5% and 97.5% quantiles of the posterior distribution of an estimate. Dead plants were excluded from analysis of end of season vegetative and generative biomass production. Differences in survival between plant types were tested by chi^2^-Tests.

## Results

### Between-type competition

**Figure 1:**
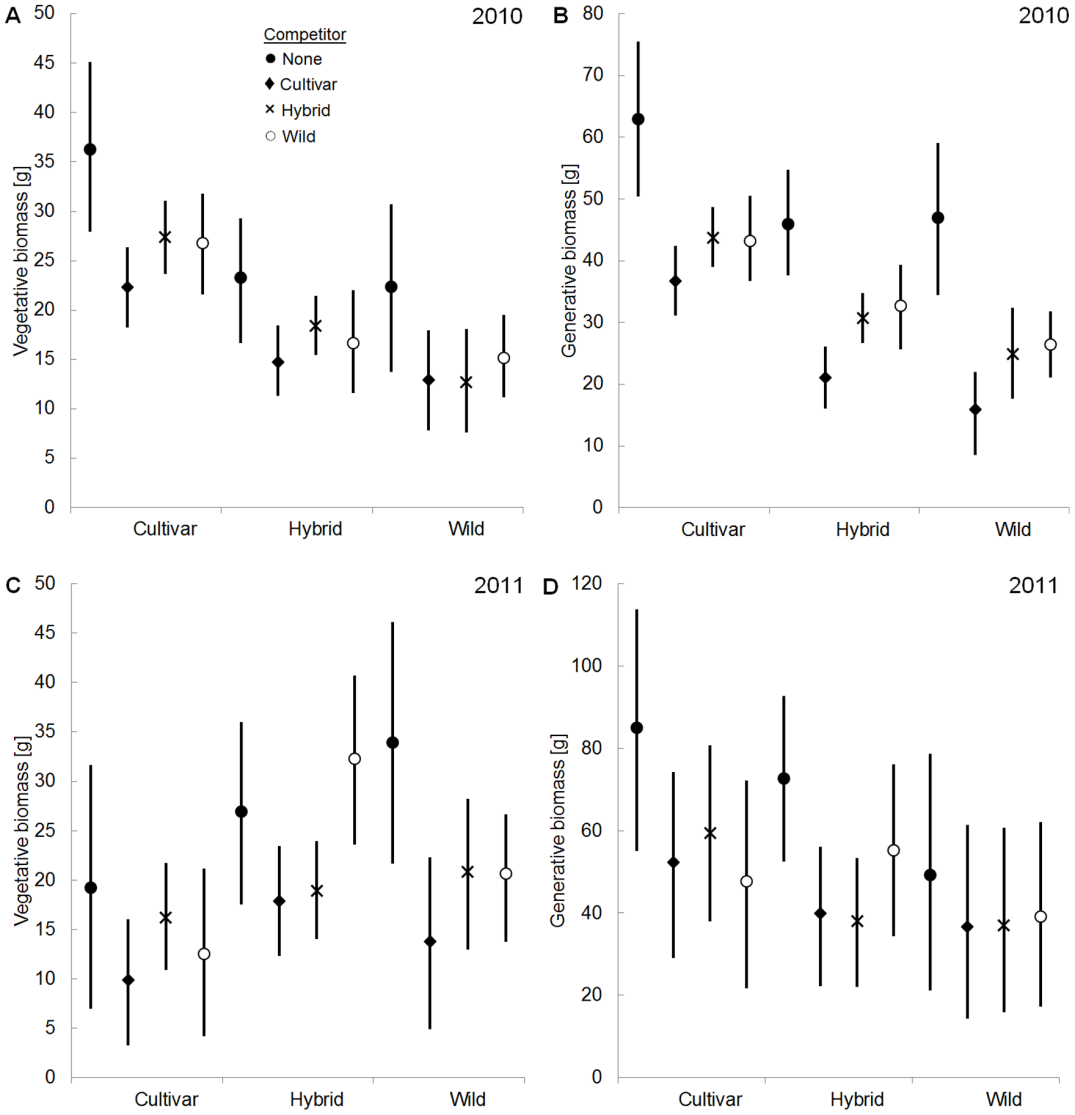
Estimated parameter means for the tested plant types of *Plantago lanceolata* in competition treatments. The figure shows biomass production of the different plant types (labels on the x-axis) grown pairwise with different competitors (see legend, competitor “none”  =  control). Vertical bars represent 95% credible intervals, based on the quantiles of the particular posterior distribution. For significance of the results, see Table 2. A) Vegetative biomass [g] in the first growing season, B) generative biomass [g] in the first growing season, C) vegetative biomass [g] in the second growing season, D) generative biomass [g] in the second growing season.

**Figure 2:**
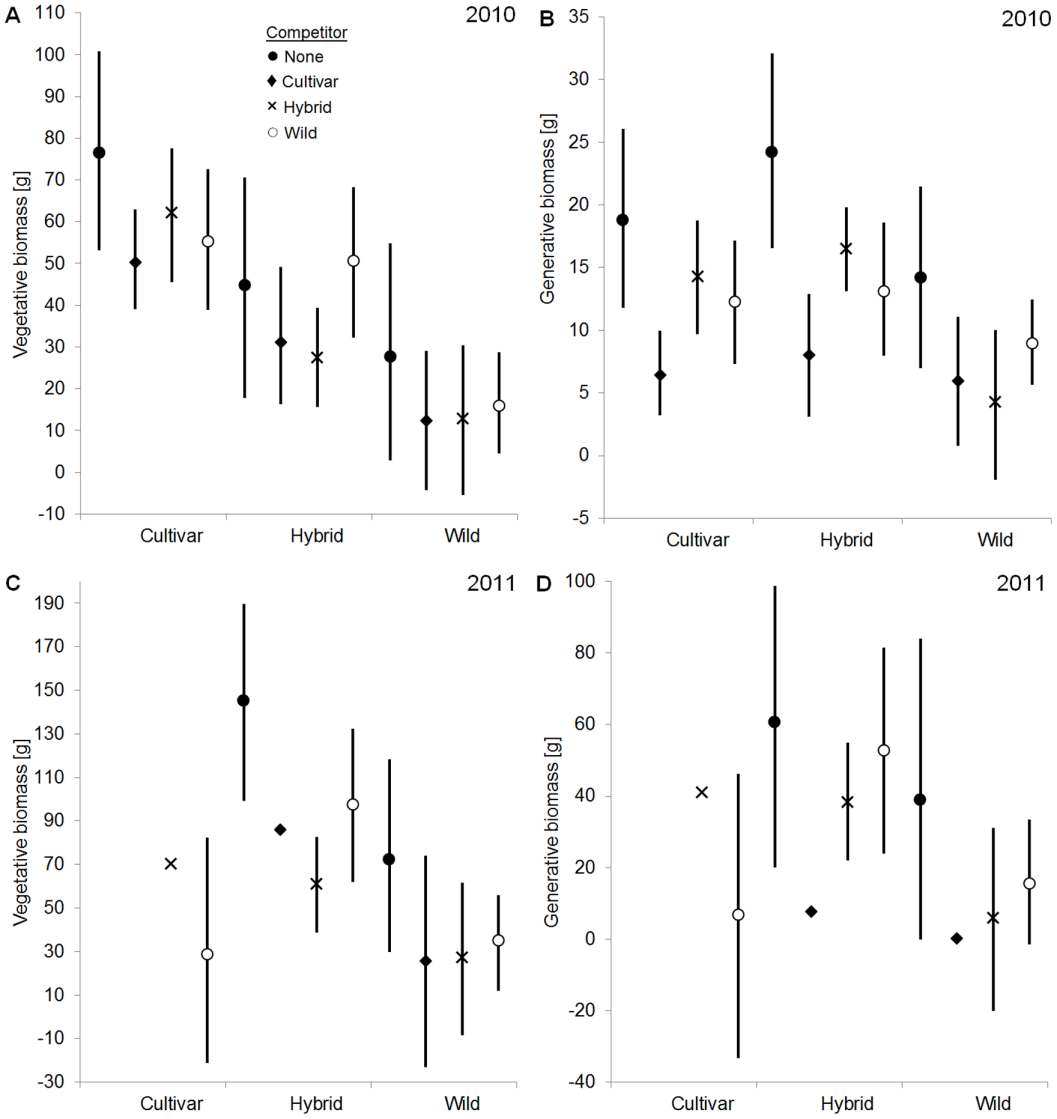
Estimated parameter means for the tested plant types of *Lotus corniculatus* in competition treatments. The figure shows biomass production of the different plant types (labels on the x-axis) grown pairwise with different competitors (see legend, competitor “none”  =  control). Vertical bars represent 95% credible intervals, based on the quantiles of the particular posterior distribution. For significance of the results, see Table 2. A) Vegetative biomass [g] in the first growing season, B) generative biomass [g] in the first growing season, C) vegetative biomass [g] in the second growing season, D) generative biomass [g] in the second growing season.

In between-type competition with wilds (Hypothesis 1), cultivars of both species produced much more vegetative and generative biomass than wilds in the first growing season. *P. lanceolata* cultivars produced 106.8% more vegetative biomass (Fig. 1A) and 171.6% more generative biomass than their wilds (Fig. 1B). The *L. corniculatus* cultivar produced 346.2% more vegetative biomass (Fig. 2A) and 106.3% more generative biomass than the wild (Fig. 2B). These differences were all highly significant (Table 2). No differences in biomass production between cultivars and wilds of *P. lanceolata* were detected in the second growing season (Figs. 1C and 1D, Table 2). In the second growing season a test for differences in biomass production between the *L. corniculatus* cultivar and wild was not conducted because of nearly complete mortality of the cultivar.

**Table 2:** Estimated differences of parameters for tested hypotheses.*

		*Plantago lanceolata*				*Lotus corniculatus*			
*Hypotheses*		*ß*	*q_2.5%_*	*q_97.5%_*	*p(ß<0) or p(ß>0)*	*ß*	*q_2.5%_*	*q_97.5%_*	*p(ß<0) or p(ß>0)*
*Vegetative biomass*									
Cultivar [wild] > wild [cultivar]	2010	13.908	6.306	21.613	**1**	42.907	20.151	67.486	**1**
	2011	−1.095	−12.987	9.808	0.559	–	–	–	–
Hybrid [wild] > wild [hybrid]	2010	4.092	−3.182	11.541	0.863	38.563	13.127	62.377	**0.997**
	2011	11.397	−0.811	22.770	**0.963**	70.900	22.204	123.064	**0.998**
Wild [wild] > wild [cultivar]	2010	2.280	−4.089	8.932	0.746	4.158	−15.201	23.343	0.651
	2011	6.963	−3.399	18.465	0.894	9.636	−47.151	60.920	0.648
Wild [wild] > wild [hybrid]	2010	2.518	−4.674	9.227	0.766	3.868	−17.336	24.482	0.65
	2011	−0.313	−10.746	10.648	0.521	7.728	−31.315	49.216	0.648
Cultivar [wild] > cultivar [cultivar]	2010	4.362	−2.527	10.829	0.891	5.370	−14.701	25.176	0.706
	2011	2.610	−7.720	13.399	0.669	–	–	–	–
Hybrid [wild] > hybrid [hybrid]	2010	−1.677	−7.856	4.161	0.708	23.665	2.259	46.094	**0.979**
	2011	13.367	3.490	22.893	**0.995**	37.003	−3.611	78.307	**0.96**
*Generative biomass*									
Cultivar [wild] > wild [cultivar]	2010	27.569	18.160	36.725	**1**	6.293	−0.877	13.449	**0.958**
	2011	10.391	−22.146	44.721	0.731	–	–	–	–
Hybrid [wild] > wild [hybrid]	2010	7.490	−2.446	17.696	0.917	9.007	1.004	17.237	**0.984**
	2011	16.829	−11.950	46.865	0.856	46.515	8.790	84.716	**0.989**
Wild [wild] > wild [cultivar]	2010	10.765	2.119	19.800	**0.996**	3.067	−3.330	9.155	0.837
	2011	2.658	−17.234	22.249	0.603	–	–	–	–
Wild [wild] > wild [hybrid]	2010	1.217	−7.541	11.068	0.6	4.699	−1.843	11.188	0.911
	2011	1.660	−16.727	20.245	0.568	9.665	−21.564	41.270	0.738
Cultivar [wild] > cultivar [cultivar]	2010	6.435	−2.381	14.873	0.911	5.739	−0.569	11.709	**0.968**
	2011	−4.822	−25.603	14.322	0.675	–	–	–	–
Hybrid [wild] > hybrid [hybrid]	2010	1.856	−6.493	9.687	0.682	−3.200	−9.383	3.059	0.842
	2011	17.491	1.073	33.412	**0.982**	14.090	−18.672	48.431	0.802

* Example for reading. Hypothesis: cultivar [wild] > wild [cultivar] means: cultivars produce more biomass in competition with wilds than wilds in competition with cultivars.

*ß*  =  estimated coefficient (mean of posterior distribution), *q_2.5%_* and *q_97.5%_* = 2.5% and 97.5% quantiles of the posterior distribution (95% credible interval), *p* = posterior probability that the estimated coefficient is smaller (for negative estimates) or larger (for positive estimates) than 0 is given. Differences in parameters with *p*≥0.95 and with 95% credible intervals that do not include or narrowly include zero are judged as significant. Significant differences are shown in bold.

During both growing seasons hybrids of both species in between-type competition with wilds (Hypothesis 2), allocated more biomass than wilds. *P. lanceolata* hybrids tended to produce more generative biomass than the wilds in the first season (+31.6%, Fig. 1B; zero was only closely within the 95% credible interval, *p* = 0.917) and significantly more vegetative biomass than competing wilds in the second growing season (+54.6%, Fig. 1C, Table 2). *L. corniculatus* hybrids produced much more vegetative (first season: +295.1%, Fig. 2A; second season: +256.9%, Fig. 2C) and generative biomass (first season: +207.3%, Fig. 2B; second season: +772.5%, Fig. 2D) than their wilds in both study years. These differences were all significant (Table 2).

### Between-type competition vs. within-type competition

Comparing between-type competition and within-type competition (Hypotheses 3 and 4) in both species, wilds produced in competition with their cultivars the same vegetative and generative biomass as wilds in competition with wilds (Figs. 1A–D and 2A–D). With the exception of *P. lanceolata*’s generative biomass production in the first season, no significant differences in biomass production between wilds in competition with cultivars and wilds in competition with wilds were detected (Table 2). In both species wilds in competition with hybrids did not produce less vegetative and generative biomass than wilds in competition with wilds (Figs. 1A–D and 2A–D, Table 2).

In both species cultivars tended to produce higher biomass in competition with wilds (between-type competition) than in competition with cultivars (within-type competition) in the first growing season (Hypothesis 5). *P. lanceolata* cultivars tended to produce 19.8% more vegetative biomass (Fig. 1A, *p* = 0.891, Table 2) and 17.7% more generative biomass (Fig. 1B, *p* = 0.911, Table 2) in competition with wilds than in competition with cultivars. There were no significant differences in the second season (Table 2). The *L. corniculatus* cultivar produced significantly more generative biomass (+91.3%) in competition with the wild than in competition with cultivar individuals in the first season (Table 2).

While *P. lanceolata* hybrids did not produce more biomass when in competition with wilds (between-type competition) than in competition with hybrids (within-type competition) in the first growing season, they did in the second growing season (Hypothesis 6). Hybrids produced considerably more vegetative (+70.9%, Fig. 1C) and generative biomass (+44.6%, Fig. 1D) when competing with wilds than in competition with hybrids in the second growing season. These differences were highly significant (Table 2). The *L. corniculatus* hybrid produced significantly more vegetative biomass in competition with the wild than in competition with hybrids in both growing seasons (first season: +84.5%, Fig. 2A; second season: +59.4%, Fig. 2C). There were no significant differences in generative biomass production in both seasons (Table 2).

### Survival

In both study species both cultivars as well as hybrids did not reduce survival of competing wilds more than wilds did in competition with wilds. In *P. lanceolata* no differences in survival between the different plant types were detected (chi^2^ = 0.543, df = 3, *p* = 0.761, Pearson's Chi-squared Test). In all plant types survival was above 90%. Although, in case of *L. corniculatus* the plant type had a significant effect on survival (chi^2^ = 32.941, df = 2, *p*<0.001), mortality was independent of neighbor presence and neighbor identity with 83% mortality for the cultivar, 8% for the wild and 4% for the hybrid.

## Discussion

### Between-type competition

Our results of between-type competition indicate that in both species cultivars are in competition with wilds at least temporarily, i.e. in the first growing season, fitter than wilds as they showed much more vegetative and generative biomass allocation. That result fits to results from restored North American tallgrass prairies [28]. However, in our study the cultivars’ advantage disappeared by the end of the second season of study. The reduced fitness in cultivars in our experiment was most likely a result of the exceptional long and cold winter conditions in 2010/2011 (31 days of temperature permanently below 0°C, about twice the no. of such days as measured as mean for the years between 1979 to 2009, www.dwd.de) rather than a result of competition, as the lower biomass production in *P. lanceolata* cultivars and the high mortality in the *L. corniculatus* cultivar was independent of competition. In our study the wilds coped better with local climatic conditions than their cultivars until the end of the experiment. In a previous study (Schröder and Prasse, unpublished data) we assumed changes in trade-offs in plant traits by cultivation. Selection towards large biomass production may in this case have included a selection against the ability to tolerate stressful environmental conditions due to changes in resource allocation. Therefore, cultivars may not be well adapted to harsh climatic conditions like frost [29]. Such trade-offs may also result in lower resistance to pest infestation [30]. Thus, cultivars may only be competitively superior as long as they are not subjected towards stressful environmental factors.

While cultivars experienced a fitness reduction in *P. lanceolata* and a high mortality in *L. corniculatus* in the second growing season, hybrids’ fitness of both species was not negatively affected. In between-type competition with their wilds hybrids tended to be fitter than wilds in both growing seasons, indicating a competitively superiority. We assume that hybrids inherited both the adaptations to the local climatic conditions from their wild parents as well as the ability to allocate high biomass from their cultivated parents. Similar inheritance of favorable life-history traits has been detected in hybrids between wild *Echinacea purpurea* and a cultivated relative [31]. In this study the larger reproductive output of hybrids in competition with their wilds was assumed to be a result of inheritance of floricultural characteristics of the parental cultivar, bred towards showy flower occurrence.

### Between-type competition vs. within-type competition

Contrary to our hypotheses 3 and 4, fitness of wilds both in competition with cultivars as well as in competition with hybrids (between-type competition) was not lower than fitness of wilds in competition with wilds (within-type competition). We interpret this finding as a strong “competitive response” [32]. The wilds are probably, at least over the short-term, able to tolerate resource reduction by competing neighbors regardless of whether they belong to the same plant type or not.

However, potential competitive superiority of cultivars over their wilds at least in the first growing season as detected in between-type competition trials (Hypothesis 1) is also indicated by greater biomass allocation in cultivars in competition with wilds than in cultivars in competition with cultivars (within-type competition). Hence, with regard to cultivars, between-type competition with wilds is lower than within-type competition in the first growing season. The tendency to competitive superiority of the hybrids over their wilds in our study was also strongly accompanied by larger fitness in competition with wilds (between-type competition) if compared to competition with hybrids (within-type competition, vegetative and generative biomass in the second season in *P. lanceolata*, vegetative biomass in both seasons in *L. corniculatus*). Therefore, in hybrids, between-type competition with wilds is lower than within-type competition in both growing seasons.

### Relevance for practice

Human selection towards vigorously growing cultivars with high biomass allocation seems to lead to a competitive superiority over wilds in the studied species. Hybrids and cultivars (at least temporarily) have the ability to suppress competing wilds (competitive effect, [32]). As between-type competition in hybrids always tended to be lower than within-type competition, from a theoretical perspective, we strongly assume that hybrids indeed have the ability to outperform their wilds potentially resulting in competitive exclusion of the latter over the long-term. However, long-term research is needed to test in-situ whether that effect leads to an outperformance of wilds and/or even other species of native vegetation.

Otherwise, it is also possible that the following generations of hybrids may suffer from hybrid breakdown (disruption of co-adapted gene complexes via recombination) due to backcrosses with parental cultivars and wilds [7]. Moreover, further evolving hybrids might inherit more maladaptive life-history traits from parental cultivars or maladaptive trade-offs than 1^st^-generation hybrids in our study [29], [30]. Such development may lead to a selection against hybrids over the long-term, assuming that natural selection is identifying over the long-term the most beneficial trade-offs and allows re-evolving towards the wild’s behavior [33]. However, re-evolution of the wild’s behavior may be countered by the vast amount of seeds from cultivars permanently introduced by re-vegetation and restoration measures. Survival and successful reproduction of hybrids as shown in our study will multiply the “seed rain” from individuals with altered life-history traits.

Our findings are not only limited because of the short study period, but also because we only tested for the competitive abilities between plants of the same age. It is known that the relative emergence times of competing species may have a significant effect on the outcome of competitive interaction. Individuals that emerge earlier may have advantages in competition for resources, because these resources are not available for the later emerging individuals [34], [35]. E.g., we detected a faster and more abundant germination (assuming less seed dormancy) in cultivars and hybrids compared to their wilds in *P. lanceolata* and *L. corniculatus* [22]. Cultivars and hybrids may therefore have an advantage over wild plants when sown at the same time or in habitats where permanent recruitments from seeds may be important for populations’ long-term existence. Whether cultivars and hybrids are able to establish over the long-term and may invade into already existing native vegetation needs again further research. As plant–age-relationships are known to play an important role in the outcome of competition [36], further studies should also focus on competition between “invading” cultivars or hybrids and established wilds as well as further species from the native vegetation.

However, the phenomenon that plant size and large biomass allocation is positively correlated with competitive superiority (especially in pairwise competition experiments) does not inevitably result in an overall dominance of such species at a plant community level [17]. In less productive and more stressful environments the aboveground biomass presumably may be not a good indicator for competitive abilities and the outcome of experiments in competition. For instance, in more arid and less trophic habitats belowground biomass may play a more important role than aboveground biomass [37]. However, also belowground biomass can be increased by cultivation of plant species used in re-vegetation [38].

## Conclusions

Despite the mentioned limitations of our study it became clear that especially hybrids between introduced cultivars and wilds are not only able to survive and reproduce under local climatic conditions but they proofed to be competitively superior to their wilds over the short-term. Thus, some traits relevant for a successful invasion are selected during cultivation and handed over into future plant generations. Although we are not able to predict the ultimate fate of cultivars and hybrids in the natural range of their wild relatives, we assume that over the long-term the risk of cryptic invasion of cultivars, especially by hybridization is high. Further studies should evaluate (e.g. by genetic structure analysis, [5], [6]) if populations of purely wild plants whose cultivars have been used in re-vegetation since several decades are still available, as there is a real threat that they are not [39].

Nevertheless, we recommend to apply the precaution principle and to avoid the use of cultivars in re-vegetation and restoration [40], [41]. If wild plant material needs to be propagated for use in re-vegetation, collection and propagation needs to follow strategies designed to reduce selection towards highly productive genotypes (both vegetative as well as generative) as competitive strength in both study species seems to be related to vigor growth with high biomass allocation. Propagation in nurseries should be done under “nearly natural” environmental conditions (e.g., avoiding irrigation and accounting for the enhanced infra-specific densities by applying only careful fertilization) and for a limited number of generations to prevent unintended selection towards certain life-history traits.
